# Dapson in heterocyclic chemistry, part VIII: synthesis, molecular docking and anticancer activity of some novel sulfonylbiscompounds carrying biologically active 1,3-dihydropyridine, chromene and chromenopyridine moieties

**DOI:** 10.1186/1752-153X-6-64

**Published:** 2012-07-02

**Authors:** Mansour S Al-Said, Mostafa M Ghorab, Yassin M Nissan

**Affiliations:** 1 Medicinal, Aromatic and Poisonous Plants Research Center (MAPPRC), College of Pharmacy, King Saud University, 2457, Riyadh, 11451, Saudi Arabia; 2 Pharmaceutical Chemistry Department, Faculty of Pharmacy, Cairo University, Cairo, Egypt

**Keywords:** Sulfone, Pyridines, Chromenes, Pyridnochromenes, Anticancer activity

## Abstract

Several new sulfonebiscompounds having a biologically active 1,2-dihydropyridine-2-one 3–19, acrylamide 20, chromene 21, 22 and chromenopyridine 23, 24 moieties were synthesized and evaluated as potential anticancer agents. The structures of the products were confirmed via elemental analyses and spectral data. The screening tests showed that many of the biscompounds obtained exhibited good anticancer activity against human breast cell line (MCF7) comparable to doxorubicin which was used as reference drug. Compounds 11, 17 and 24 showed IC_50_ values 35.40 μM, 29.86 μM and 30.99 μM, respectively. In order to elucidate the mechanism of action of the synthesized compounds as anticancer agents, docking on the active site of farnesyltransferase and arginine methyltransferase was also performed and good results were obtained.

## Background

Many naturally occurring and synthetic compounds containing the 2-pyridone scaffold possess interesting pharmacological properties [1]. The pyridine derivative I, for example, has been identified as specific non-nucleuoside reverse transcriptase inhibitor in treatment of HIV-1 [2,3]. While the pyridine derivatives, Milirinone II and Amrinone III, and their analouges are used as cardiotonic agents in the treatment of heart failure [4-7]. Also, Pirfeidione (PFD) IV, a pyridine derivative which demonstrated antifibrotic activity in several organs in experimental animals, including lung, kidney and uterus has proven beneficial cure for a range of fibrotic conditions through both anti-inflammtory and and antifibrotic mechanisms [8]. A phase II clinical study showed PFD to be promising agent for the treatment of idiopathic pulmonary fibrosis, initiated in mice treated with cyclophosamide [9], amiodarone [10] or belomycin [11-16]. The reported antifbrotic activity of PFD prompted us to synthesize a new series of sulfonebiscompounds carrying biologically active 1,2-dihydropyridine-2-one, chromene and chromenopyridine as analoges to PFD. In addition, some 2-pyridones are also reported to possess antitumor, antibacterial [17] and other biological activities [18-20]. On the otherhand, sulfone derivatives have been found to exhibit a wide variety of pharmacological activities [21-25]. In addition, the bisheterocyclic compounds chromenes and chromenopyridine derivatives are well known as anticancer agents [26-29]. Also, diphenylsulfones and bisheterocyclic compounds are reported to have a broad spectrum of biological activities. Some are endowed with antitumor or antifungal properties [30]. On the other hand, some pyridine and isoquinoline derivatives have various biological properties such as antimicrobial [31], anticancer [32-35] activities.

Recent studies have proved the remarkable effect of Dapson on inhibiting cell growth in glioblastoma by acting as anti-VEGF and anti-angiogenic agent via depriving glioblastoma of neutrophil-mediated growth promoting effects [36]. Allantodapson V, a Dapson derivative showed high activity as anticancer through inhibition of arginine methyltranseferase (PRMT1) an enzyme which plays an important role in hormone dependent cancers. A series of acylated diarylsulfone derivatives were evaluated for the same activity and compound VI exihibited good activity as (PRMT1) inhibitor [37].

In view of these findings, and in continuation to our work in the synthesis of novel anticancer agents [38-42] we undertook the synthesis of bisheterocyclicsulfone compounds analogues for 2-pyridones incorporating biologically active 1,2-dihydropyridine-2-one, chromene, and chromenopyridone in one molecule to explore the promising anticancer compounds.

## Results and discussion

### Chemistry

**Scheme 1:**
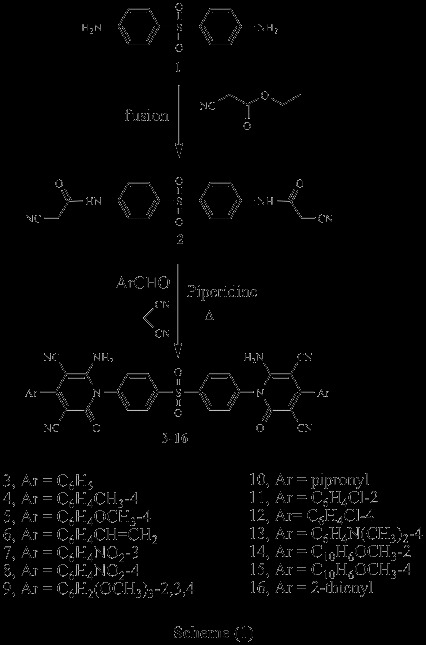
Synthetic pathways used to obtain compounds 2-16.

**Scheme 2:**
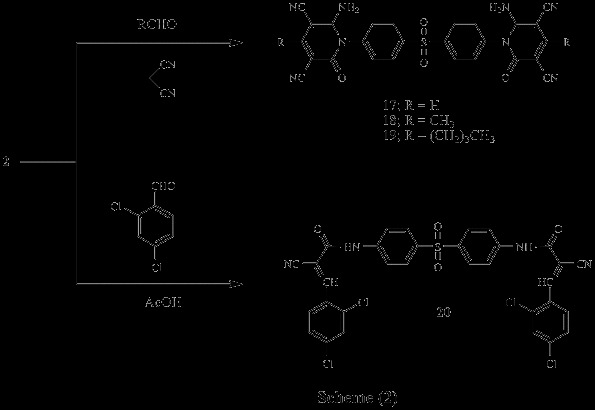
Synthetic pathways used to obtain compounds 17-20.

**Scheme 3:**
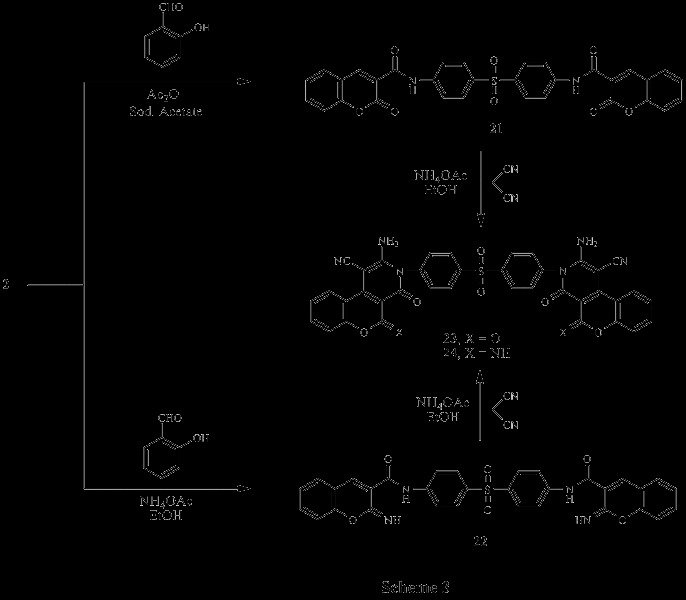
Synthetic pathways used to obtain compounds 21-24.

Several compounds were designed with the aim of exploring anticancer properties (Scheme 1, Scheme 2, Scheme 3). Scheme 1 outlines the synthetic pathway used to obtain compounds 3–16. The starting material N,N’-(4,4’-sulfonylbis(4,1-phenylene))bis(2-cyanoacetamid) 2 was obtained via reaction of Dapson 1 with ethyl cyanoacetate. Compound 2 was established by elemental analysis and spectral data. Thus, IR spectrum of 2 revealed bands at 3448, 3363 cm^-1^ (2NH), 2256 cm^-1^ (2 C ≡ N), 1701 cm^-1^ (2 C = O) and 1342, 1180 cm^-1^ (SO_2_). ^1^ H-NMR spectrum of 2 in (DMSO-d_6_) exhibited signals at 4.0 ppm due to CH_2_ group, 7.4-7.9 ppm corresponding to aromatic protons and 10.7 ppm due to 2 NH groups. Treatment of compound 2 with appropriate aldehyde and malononitrile in the presence of catalytic amounts of pipredine compounds 3–16, respectively. These compounds were verified on the basis of elemental analyses, IR, ^1^ H-NMR and ^13^ C-NMR. Thus, IR spectra of compounds 3–16 exhibited the presence of NH_2_, C ≡ N, C = O and SO_2_ bands. ^1^ H-NMR spectra of compounds 3–16 in (DMSO-d_6_) revealed the presence of NH2 at 6.0-6.8 ppm and aromatic protons at 6.9-8.7 ppm (Scheme 1).

Similarly, interaction of 2 with aliphatic aldehyde and malononitrile in ethanol containing catalytic amount of piperidine afforded the corresponding 1,2-dihydropyridine-2-one derivatives 17–19. IR spectra of compounds 17–19, exhibited the presence of characteristic bands of NH_2_, C ≡ N, C = O and SO_2_ groups ^1^H-NMR spectrum of 17 in (DMSO-d_6_) revealed signals at 6.8 ppm due to 2NH_2_, while ^1^ H-NMR spectrum of 18 in (DMSO-d_6_) revealed signals at 1.8 ppm corresponding to 2CH_3_. On the other hand, ^1^ H-NMR spectrum of 19 in (DMSO-d_6_) triplet signal at 1.1 ppm for CH_3_ and a multiplet one at 1.3-2.0 ppm corresponding to CH_2_ groups. Interaction of 2 with 2,4-dichlorobenzaldehyde in acetic acid gave the corresponding acrylamide derivative 20. IR spectrum of 20 revealed bands at 3372 cm^-1^ (2NH), 2203 cm^-1^ (2 C ≡ N), 1652 cm^-1^ (2 C = O) and 829 cm^-1^ (C-Cl). ^1^ H-NMR spectrum of 20 in (DMSO-d_6_) showed signals at 8.0 ppm due to CH groups, 10.0 ppm corresponding to NH groups (Scheme 2).

Furthermore, Perkin reaction was carried out by reacting compound 2 with salicylaldehyde in acetic anhydride containing catalytic amount of anhydrous sodium acetate to give the corresponding chromene derivative 21, while reaction of 2 with salicylaldehyde in ammonium acetate afforded 2-iminochromene derivative 22 (Scheme 3).

### Molecular docking

The zinc-metalloenzyme farnesyl transferase (FTase) catalyzes the transfer of a farnesyl group to a cysteine thiol group contained in the C-terminal tetra peptide signal sequence of Ras, frequently referred to as aCAAX motif. Farnesylation causes membrane localization of Ras which, in turn, determines the switch from an inactive to an active Ras-GTP-bound form [43-45]. Among the Ras isoforms H-ras, N-ras, and K-ras, mutations in the K-ras isoform are most relevant to human cancers in particular pancreatic, colon, and lung cancers, which exhibit approximately 90, 40, and 25% incidence of Kras mutations, respectively. Inhibitors of FTase prevent membrane localization of the Ras oncogene and have the ability to revert the transformed phenotype, providing the rationale for the development of farnesyl transferase inhibitors (FTIs) as anticancer drugs [46-49].

On the other hand, the relative levels of arginine methyltransferase (PRMT1) isoforms are altered between normal and cancerous breast issue, with two of the isoforms down-regulated [50]. Therefore, it appears that PRMT1expression in cancer cells may be altered depending on the tumor type. Studies are beginning to examine the specific role of PRMT1in cancer. PRMT1 is an essential component of a Mixed Line age Leukaemia (MLL) transcriptional complex that modifies histones by methylation, at H4R3, and acetylation [51]. This serves as the first demonstration of a direct role for PRMT1-mediated transcriptional up regulation during cancer progression.

Thus, the present investigation is concerned with the synthesis of novel anticancer agents and trying to understand their mechanism of action. In order to perform the aim of the present investigations the authors have performed molecular docking of the synthesized compounds on the active sites of both farnesyl transferase and arginine methyltransferase (PRMT1) which may lead to understanding of their effect as antitumor agents.

### Molecular docking on the active site of farnesyl transferase

The protein data bank file (PDB:3E30) was selected for this purpose. The file contains farnesyl transferase enzyme co-crystallized with a sulfone ligand. All docking procedures were achieved by MOE (Molecular Operating Environment) software 10.2008 provided by chemical computing group, Canada. Docking on the active site of farnesyl transferase enzyme was performed for all synthesized compounds 2–24.

**Figure 1:**
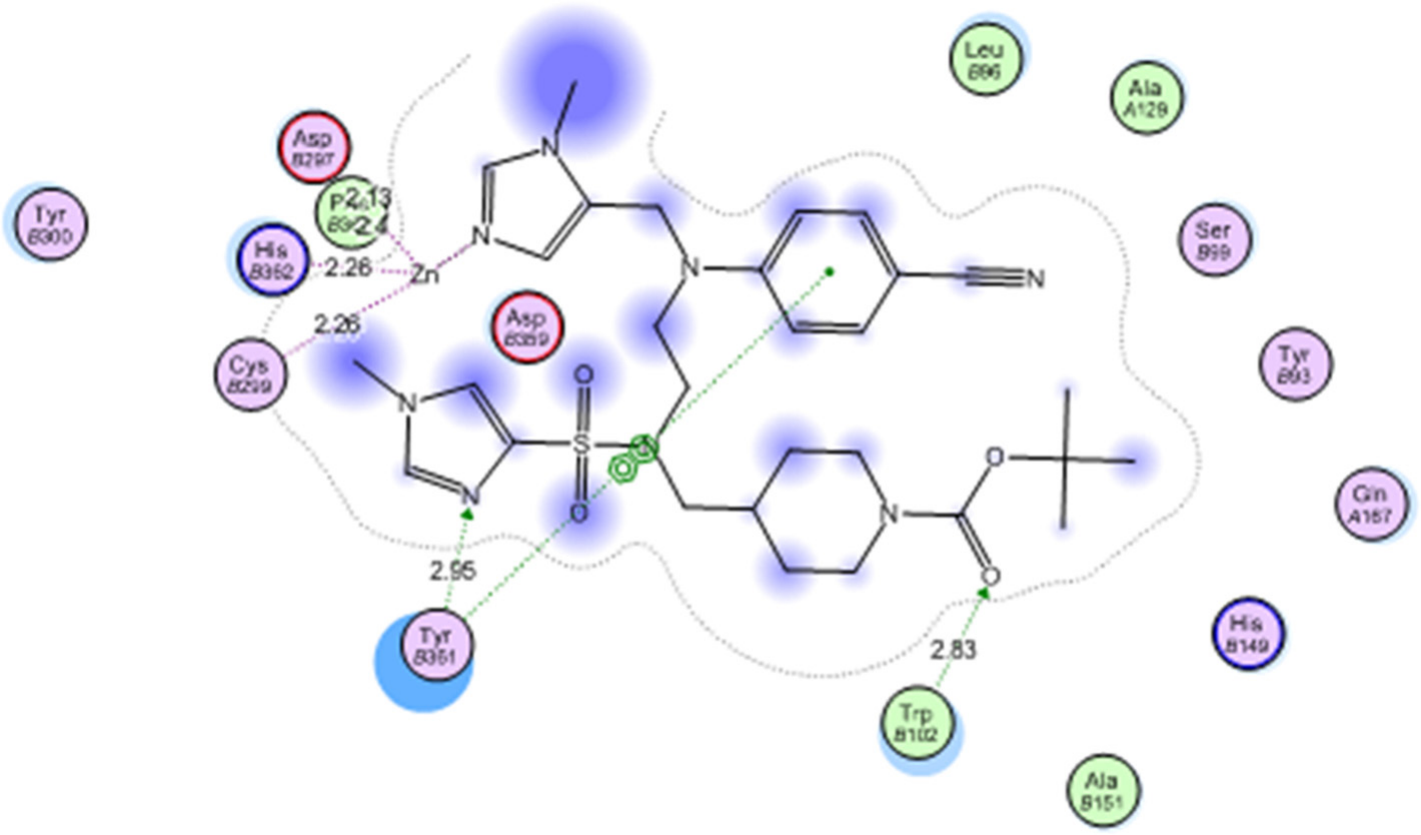
Co-crystallized sulfone ligand on the active site of farnesyltransferase.

Docking protocol was verified by redocking of the co-crystallized ligand in the vicinity of the active site of the enzyme with energy score (S) = −25.6345 Kcal/ mol and root mean standard deviation (RMSD) = 2.8268 (Figure 1).

The sulfone ligand interacts with the active site of farnesyl transferase by four interactions: Try B361 with a hydrogen bond of 2.95 A^o^ and arene-arene interaction, Trp 102 with a hydrogen bond of 2.83 and with Zn by the lone pair of imidazole nitrogen. All synthesized compounds were fit to the active site of farnesyl transferase enzyme with good energy scores (S) suggesting activity as farnesyl transferase inhibitors. Energy scores (S) and amino acid interactions for synthesized compounds were listed in (Table 1).

**Table 1:** Binding scores and amino acid interactions of the docked compounds on the active site of farnesyltransferase (FT)

**Compound no.**	**S Kcal/Mol**	**Amino acid interactions**	**H bond length A**^**o**^	**Interaction with Zn**
2	-22.2685	Leu B295, Lys B294	3.37, 2.76	No interaction
3	-37.4155	Lys A164, Arg B202	3.39, 2.53-3.14	CN
4	-25.1368	Lys B234, Tyr B334, LysB358, Arg B202	3.08, 2.75, 3.28, 2.73	SO_2_
5	-22.9916	Lys B294, Lys A164, Gln A167, Arg B202	3.47, 2.84, 3.00, 3.09	C = O
6	-31.4218	Lys A164, Arg B202	2.49, 3.28	SO_2_
7	-30.3616	Arg B291, Arg B202	3.19, 2.47-2.96	CN
8	-26.5141	Arg B291, Lys B294	3.55, 2.94	CN
9	-25.5855	Lys B294, Lys A168, His B362	2.58, 2.76, 3.19	CN
10	-27.1374	Ser B99, Ser B367, Arg B291	3.30, 3.05, 2.56	No interaction
11	-23.4085	Trp B102, Lys A168	2.75, 2.80	C = O
12	-28.7413	Lys A164, Ser B99	3.00, 3.25	CN
13	-27.1676	Lys A164, Arg B202	2.48, 2.76	SO_2_
14	-28.8232	Lys A164, Arg B202	2.81, 2.89-3.25	C = O
15	-32.2519	Tyr B300, Asn A165	3.10, 3.32	CN
16	-38.0536	Arg B202, Arg B291, Lys B294	2.57, 3.01, 3.39	CN
17	-19.9521	Lys B353, Gly B290, Lys B294, Arg B202	2.78, 3.29, 2.67, 3.13	No interaction
18	-23.0290	Leu B295, Lys B294	3.05, 2.61	No interaction
19	-32.9232	Arg B291	3.81	CN
20	-24.4073	Arg B202	2.35	C = O
21	-29.7807	Tyr B300	2.85	C = O
22	-38.6191	Arg B202, Asp B352	2.92, 1.96	C = O, NH
23	-38.8898	Lys A164, Arg B202	2.81, 2.49-2.55	C = O, C = O
24	-45.9317	Lys A164, Arg B202	2.83, 2.46-2.45	C = O, NH

**Figure 2:**
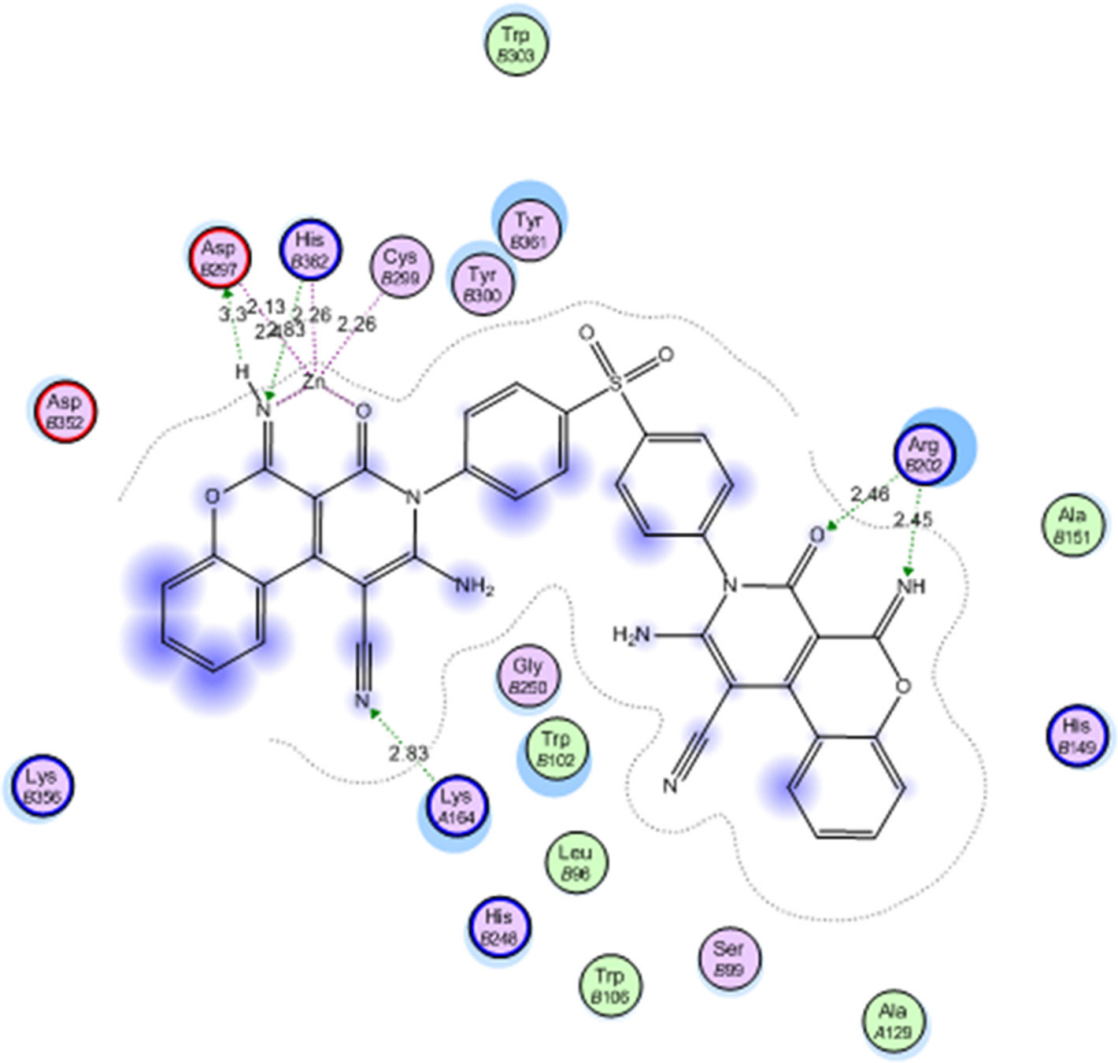
Compound 24 on the active site of farnesyltransferase.

Compound 24 showed the best energy score (S) = −45.9317 Kcal/mol and interacted with Lys A146 with a hydrogen bond of 2.83 A^o^, with Arg B202 with two hydrogen bonds of 2.45, 2.46 A^o^ and with Zn through its C = O and NH (Figure 2).

### Molecular docking on the active site of arginine methyltransferase (PRMT1)

The protein data bank file (PDB:3Q7E) was selected for this purpose. The file contains arginine methyltransferase co-crystallized with its ligand (S-adenosyl methionine). All docking procedures were achieved by MOE (Molecular Operating Environment)software 10.2008 provided by chemical computing group, Canada. Docking on the active site of arginine methyltransferase enzyme was performed for all synthesized compounds 2–24.

**Figure 3:**
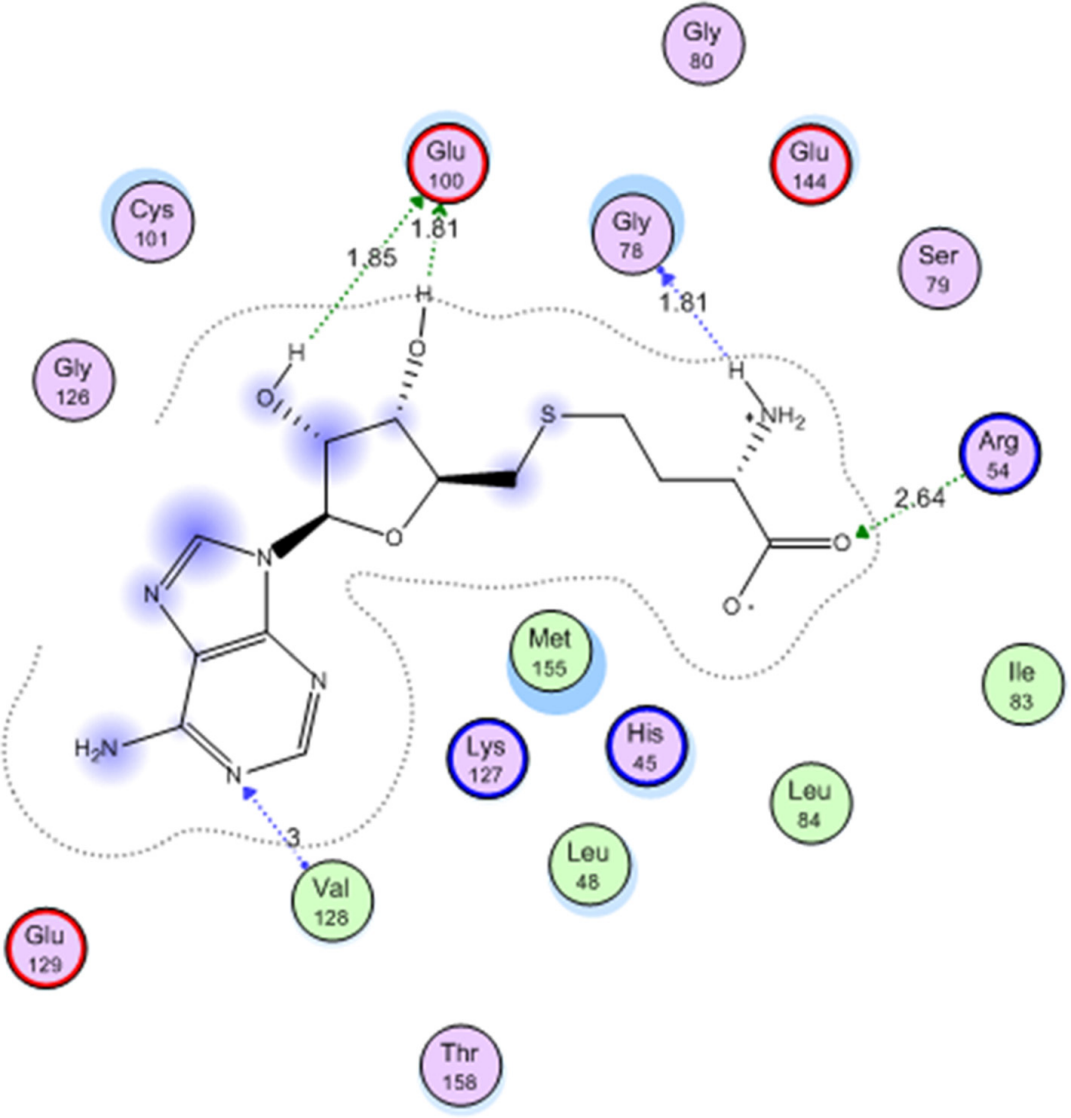
Co-crystallized S-adenosyl methionine ligand on the active site of arginine methyltransferase (PRMT1).

Docking protocol was verified by redocking of the co-crystallized ligand in the vicinity of the active site of the enzyme with energy score (S) = −18.5932 Kcal/ mol and root mean standard deviation (RMSD) = 0.3523. The ligand interacts with the active site of arginine methyltransferase by five interactions: Val 128 with a hydrogen bond of 3.00 A^o^, with Arg 54 with a hydrogen bond of 2.64, with Gly 78 with a hydrogen bond of 1.81 A^o^ and with Glu 100 with two hydrogen bonds of 181, 186 A^o^ (Figure 3).

All synthesized compounds were fit to the active site of arginine methyltransferase enzyme with good energy scores (S) except compounds 7, 18 and 19 suggesting good activity as arginine methyltransferase inhibitors for most of the synthesized compounds. Energy scores (S) and amino acid interactions for the synthesized compounds were listed in (Table 2).

**Table 2:** Binding scores and amino acid interactions of the docked compounds on the active site of arginine methyltransferase (PRMT1)

**Compound no.**	**S Kcal/Mol**	**Amino acid interactions**	**H bond length A**^**o**^
2	-20.0584	Lys 127, His 293	2.65, 2.81
3	-13.8464	Lys 127, Arg 327	2.39, 2.96
4	-17.2063	Lys 127, Arg 327	2.42-2.39, 2.45
5	-13.6909	Lys 127, His 45, Arg 327	2.57, 2.95, 2.36
6	-18.0294	Arg 327	2.45-3.02
7	11.0959	------------	------------
8	-15.9006	Lys 127, Arg 327	2.40, 2.30
9	-5.1052	His 45, Glu 153, Arg 327	2.75, 1.65, 2.36
10	-17.1347	Lys 127, Glu 153, His 45	2.75, 1.58, 2.87
11	-12.0837	Asn 167	2.65
12	-19.6261	Lys 127, Arg 327	2.59-2.84, 2.85
13	-15.7402	Lys 127, Glu 153, Arg 327	2.47, 1.93, 2.44
14	-20.4078	Asn 157, Lys 127	3.18, 2.66-2.79
15	-18.8629	Gln 163, Lys 127	2.22, 2.42-3.23
16	14.8212	------------	------------
17	-20.6494	Asn 157, His 45, Lys 127	3.24, 3.21, 2.68
18	6.1835	------------	------------
19	10.1989	------------	------------
20	-17.2838	Lys 127	2.51
21	-17.6535	Lys 127	2.51, 2.86
22	-15.4395	Arg 327, Glu 144	2.79, 1.47
23	-19.4615	Lys 127	2.54, 2.52
24	-23.0582	Arg 327, Lys 127, Glu 130	2.51-2.46, 2.75, 1.36

**Figure 4:**
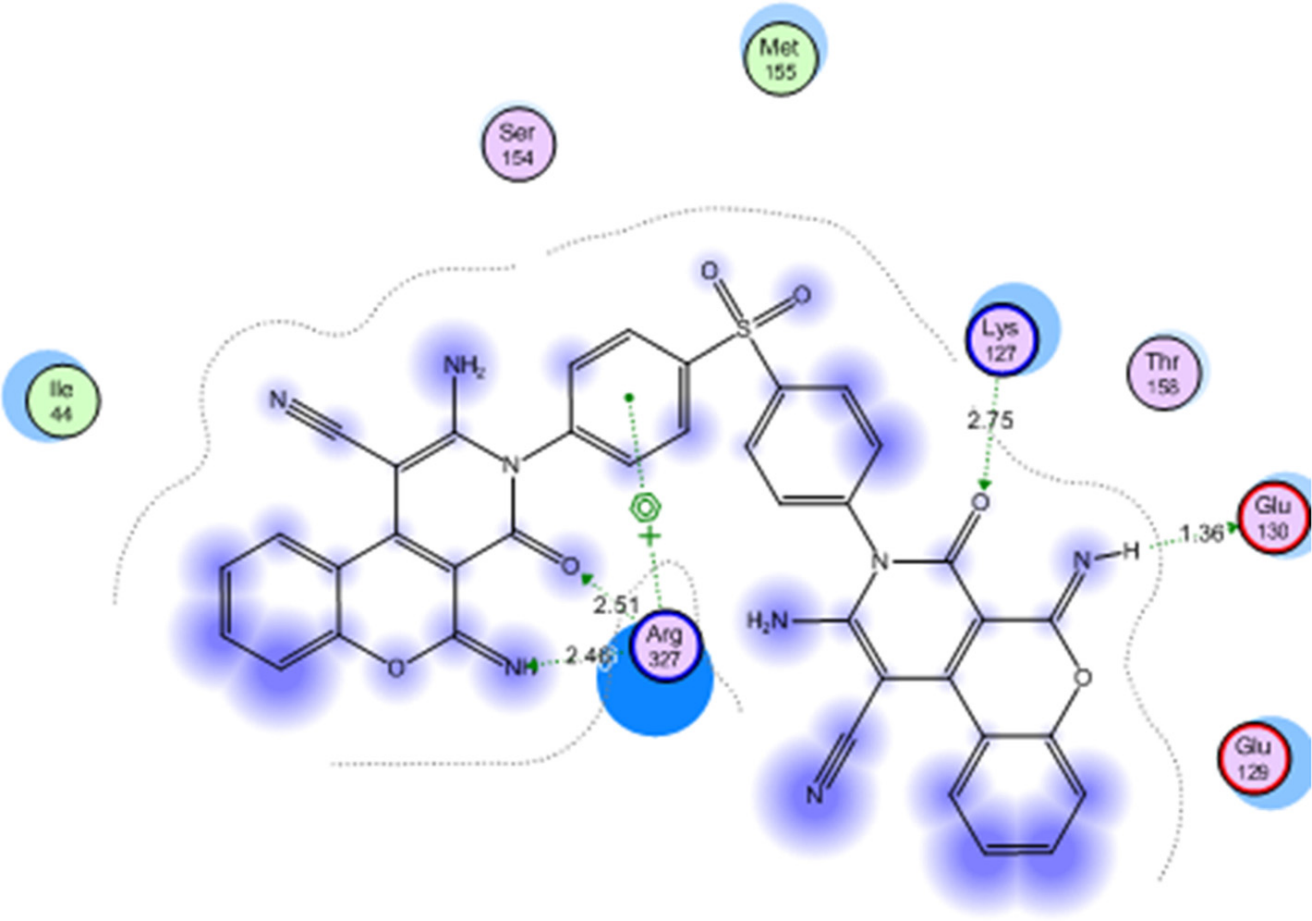
Compound 24 on the active site of arginine methyltransferase (PRMT1).

Compound 24 showed the best energy score (S) = −23.0582 Kcal/mol and interacted with Arg 327 with two hydrogen bonds of 2.51, 2.46 A^o^, with Lys 127 with a hydrogen bond of 2.75 A^o^ and with Glu 130 with a hydrogen bond of 1.36 A^o^ (Figure 4).

### *In vitro* antitumor activity

The newly synthesized compounds were evaluated for their *in vitro* cytotoxic activity against human breast cancer cell line; MCF7. Doxorubicin which is one of the most effective anticancer agents was used as the reference drug in this study. The relationship between surviving fraction and drug concentration was plotted to obtain the survival curve of breast cancer cell line (MCF7).The response parameter calculated was the IC_50_ value, which corresponds to the concentration required for 50% inhibition of cell viability. Table 3 shows the *in vitro* cytotoxic activity of the synthesized compounds where all compounds exhibited significant activity compared to the reference drug.

**Table 3:** *In vitro* anticancer screening of the synthesized compounds against human breast cell line (MCF7)

**Comp NO.**	**Compound concentration (μM)**				**IC**_**50 **_**(μM)**
	**10 μM**	**25 μM**	**50 μM**	**100 μM**	
	Surviving fraction (Mean ± S.E.)^*^				
Doxorubicin	0.721 ± 0.02	0.546 ± 0.02	0.461 ± 0.01	0.494 ± 0.03	71.80
2	0.727 ± 0.134	0.427 ± 0.055	0.307 ± 0.029	0.317 ± 0.021	46.57
3	0.793 ± 0.055	0.454 ± 0.097	0.292 ± 0.008	0.332 ± 0.050	52.45
4	0.840 ± 0.063	0.435 ± 0.035	0.403 ± 0.015	0.335 ± 0.082	54.37
5	0.906 ± 0.021	0.642 ± 0.059	0.428 ± 0.038	0.547 ± 0.046	81.22
6	0.732 ± 0.333	0.584 ± 0.046	0.406 ± 0.069	0.229 ± 0.097	49.65
7	0.761 ± 0.190	0.546 ± 0.123	0.254 ± 0.031	0.297 ± 0.048	47.83
8	0.830 ± 0.124	0.399 ± 0.082	0.199 ± 0.021	0.272 ± 0.005	42.56
9	0.649 ± 0.028	0.394 ± 0.339	0.207 ± 0.027	0.261 ± 0.049	37.29
10	0.609 ± 0.059	0.479 ± 0.095	0.332 ± 0.058	0.316 ± 0.064	45.45
11	0.747 ± 0.197	0.359 ± 0.052	0.153 ± 0.020	0.189 ± 0.002	35.40
12	0.604 ± 0.075	0.232 ± 0.019	0.376 ± 0.089	0.312 ± 0.029	40.12
13	0.650 ± 0.184	0.401 ± 0.016	0.253 ± 0.021	0.401 ± 0.017	45.77
14	0.875 ± 0.066	0.580 ± 0.046	0.336 ± 0.049	0.467 ± 0.047	65.58
15	0.886 ± 0.047	0.423 ± 0.024	0.259 ± 0.054	0.389 ± 0.047	52.48
16	0.669 ± 0.114	0.539 ± 0.088	0.276 ± 0.064	0.259 ± 0.080	44.62
17	0.509 ± 0.235	0.230 ± 0.139	0.300 ± 0.134	0.279 ± 0.065	29.86
18	0.865 ± 0.057	0.615 ± 0.048	0.232 ± 0.046	0.286 ± 0.071	50.74
19	0.815 ± 0.042	0.545 ± 0.109	0.264 ± 0.044	0.336 ± 0.096	51.48
20	0.703 ± 0.189	0.427 ± 0.194	0.251 ± 0.026	0.374 ± 0.085	46.26
21	0.941 ± 0.020	0.472 ± 0.209	0.199 ± 0.090	0.278 ± 0.108	47.49
22	0.653 ± 0.291	0.574 ± 0.180	0.337 ± 0.116	0.359 ± 0.044	52.74
23	0.878 ± 0.032	0.563 ± 0.065	0.276 ± 0.031	0.389 ± 0.058	56.37
24	0.648 ± 0.329	0.280 ± 0.154	0.174 ± 0.105	0.194 ± 0.065	30.99

* Each value is the mean of three values ± Standard Error.

All the synthesized compounds showed better cytotoxic activity than Doxorubicin except compound 5 which showed IC_50_ value 81.22 μM. The 1,2-dihdropyridine-2-one derivatives 3–19 showed IC_50_ values in the rang 29.86-81.22 μM. Compound 17 which showed IC_50_ value 29.86 μM was the most active compound. Compound 17 also showed good scoring energy S = −19.9521 kcal/Mol. and the good amino acid interactions upon docking on the active site of farnesyl transferase enzyme. It also showed good energy score S = −20.9464 kcal/Mol. and good amino acid interactions upon docking on the active site of arginine methyl transferase enzyme. Upon substitution on position 4 of compound 17 with several substitutions the activity drops. However, 2,3,4-trimethoxy phenyl substitution, 2-chloro phenyl substitution and 4-chloro phenyl substitution did not decrease the activity in the same way substitution with 4-CH_3_ phenyl, 4-OCH_3_ phenyl and 2-OCH_3_ naphthyl did. This was clearly illustrated by the values of IC_50_ of the 1,2-dihdropyridine-2-one derivatives 9, 11 and 12 with IC_50_ values of 37.29 μM, 35.40 μM and 40.12 respectively. On the other hand, the IC_50_ values for the 1,2-dihdropyridine-2-one derivatives in which the substitution was with 4-CH_3_ phenyl, 4-OCH_3_ phenyl and 2-OCH_3_ naphthyl were much higher indicating less activity. This was clearly shown in the 1,2-dihydropyridine derivatives 4,5 and 14 with IC_50_ values of 54.37 μM, 81.22 μM and 65.58 μM, respectively.

Compounds 20–24 showed cytotoxic activity with IC_50_ values in the range of 30.99 to 56.37 μM with cytotoxic activity better than that of Doxorubicin. The chromenopyridine derivative 24 was with the best IC_50_ = 30.99 μM among these compounds while compound 23 showed the highest IC_50_ value 56.37 μM among these compounds. Compound 24 also showed the best scoring energy S = −45.9317 kcal/Mol. and the best amino acid interactions upon docking on the active site of farnesyl transferase enzyme. It also showed the best energy score S = −23.0582 kcal/Mol. and the best amino acid interactions upon docking on the active site of arginine methyl transferase enzyme.

The promising results of cytotoxic activity of the synthesized compounds especially compounds 17, 24 urge more investigations for their mechanism of action. The trial in the present investigation to predict an assumption of the mechanism of action of the synthesized compounds was conducted through molecular docking on the active site of two enzymes based on the similarities between the synthesized compounds and the enzyme inhibitors of these enzymes.

### Experimental

#### Chemistry

Melting points (°C, uncorrected) were determined in open capillaries on a Gallenkemp melting point apparatus (Sanyo Gallenkemp, Southborough, UK) and were uncorrected. Precoated silica gel plates (silica gel 0.25 mm, 60 G F254; Merck, Germany) were used for thin layer chromatography, dichloromethane/methanol (9.5:0.5) mixture was used as a developing solvent system and the spots were visualized by ultraviolet light and/or iodine. Infra-red spectra were recorded in KBr discs using IR-470 Shimadzu spectrometer (Shimadzu, Tokyo, Japan). NMR spectra (in DMSO-d6) were recorded on Bruker AC-300 Ultra Shield NMR spectrometer (Bruker, Flawil, Switzerland, δ ppm) at 300 MHz using TMS as internal Standard and peak multiplicities are designed as follows: s, singlet; d, doublet; t, triplet; m, multiplet. Elemental analyses were performed on Carlo Erba 1108 Elemental Analyzer (Heraeus, Hanau, Germany).

##### N,N'-(4,4'-sulfonylbis(4,1-phenylenebis (2-cyanoacetamide) 2

A mixture of Dapsone (2.48 g, 0.01 mol.) and ethyl cyanoacetate (1.13 g, 0.01 mol.) was refluxed for 3 h, concentrated and cooled. The obtained solid was filtered and crystallized from ethanol to give 2. Yield 92%, melting point 137.5-139°C. IR:υ_max_./cm^-1^ 3448, 3363 (2 NH), 3062 (CH aromatic), 2960, 2931 (CH aliphatic), 2256 (CN), 1701 ( 2 C = O),1342, 1180 (SO_2_). ^1^ H-NMR (DMSO-d_6,_ D_2_O):δ 4.0 (s, 4 H, 2CH_2_), 7.4-7.9 (m, 8 H, Ar-H), 10.7 (s, 2 H, 2NH exch.). ^13^ C-NMR(DMSO-d_6,_ D_2_O): 24.4(2), 115.6(2), 119.2(2), 119.3(2), 128.1(2), 129.2(2), 137.8(2), 142.7(2), 162.2(2). Anal. Calcd. for C_18_H_14_N_4_O_4_S (382.39): C, 56.54; H, 3.69; N, 14.65. Found: C, 56.81; H, 3.84; N, 14.29.

#### General procedure for compounds 3–16 and 17–19

A mixture of the starting material 2 (6.86 g, 0.01 mol.), appropriate aldehydes (0.01 mol.) and malononitrile (0.66 g, 0.01 mol.) in ethanol (50 mL) containing catalytic amount of piperidine in ethanol (50 mL) was heated under reflux for 5 h. The obtained solid was crystallized from dioxane to give 3–19, respectively.

##### 1,1'-(4,4'-sulfonylbis(4,1-phenylene))bis(6-amino-2-oxo-4-phenyl-1,2-dihydropyridine-3,5-dicarbonitrile) 3

Yield 68%, melting point 257.7-259°C. IR:υ_max_./cm^-1^ 3448, 3371 (2 NH_2_), 3077 (CH aromatic), 2210 (2 C ≡ N), 1670 (2 C = O),1290 (2 C = S), 1399, 1149 (SO_2_).^1^ H-NMR (DMSO-d_6,_ D_2_O):δ 6.2 (s, 4 H, 2NH_2_, exch.), 7.4-7.9 (m, 18 H, Ar-H). Anal. Calcd. for C_38_H_22_N_8_O_4_S(686.70): C, 66.46; H, 3.23; N, 16.32. Found: C, 66.71; H, 3.20; N, 16.00.

##### 1,1'-(4,4'-sulfonylbis(4,1-phenylene))bis(6-amino-2-oxo-4-p-tolyl-1,2-dihydropyridine-3,5-dicarbonitrile) 4

Yield 76%, melting point 234.2°C. IR:υ_max_./cm^-1^ 3371, 3209 (2 NH_2_), 3100 (CH aromatic), 2960, 2870 (CH aliphatic),2218 (2 C ≡ N), 1674 (2 C = O), 1400, 1149 (SO_2_). ^1^ H-NMR (DMSO-d_6,_ D_2_O):δ 2.4 (s, 6 H, 2 CH_3_), 6.6 (s, 4 H, 2NH_2_, exch.), 7.3-8.1 (m, 16 H, Ar-H). ^13^ C-NMR(DMSO-d_6,_ D_2_O): 17.6 (2), 80.2 (2), 114.2 (2), 115.6 (4), 128.0 (4), 128.6 (4), 130.1 (4), 131.0 (4), 133.8 (4), 134.7 (4), 152.8 (2), 153.9 (2), 163.4 (2). Anal. Calcd. forC_40_H_26_N_8_O_4_S(714.75): C, 67.22; H, 3.67; N, 15.68. Found: C, 67.56; H, 3.44; N, 15.50.

##### 1,1'-(4,4'-sulfonylbis(4,1-phenylene))bis(6-amino-4-(4-methoxyphenyl)-2-oxo-1,2-dihydropyridine-3,5-dicarbonitrile) 5

Yield 71%, melting point >340°C.IR:υ_max_./cm^-1^ 3390, 3210 (NH, NH_2_),3100 (CH aromatic), 2962, 2839 (CH aliphatic), 2214 (2 C ≡ N), 1680 (2 C = O), 1400, 1180 (SO_2_).^1^ H-NMR (DMSO-d_6,_ D_2_O):δ 3.8 (s, 6 H, 2 OCH_3_), 6.3 (s, 4 H, 2NH_2_, exch.), 7.0-8.1 (m, 16 H, Ar-H). ^13^ C-NMR(DMSO-d_6,_ D_2_O): 55.2 (2), 77.9 (2), 113.7 (4), 117.6 (2), 117.9 (4), 127.5 (4), 129.1 (2), 129.5 (4), 130.8 (4), 140.6 (2), 141.2 (2), 157.7 (2), 159.6 (2), 160.3 (2), 160.8 (2). Anal. Calcd. forC_40_H_26_N_8_O_6_S(746.75): C, 64.34; H, 3.51; N, 15.01. Found: C, 64.24; H, 3.19; N, 15.38.

##### 1,1'-(4,4'-sulfonylbis(4,1-phenylene))bis(6-amino-2-oxo-4-styryl-1,2-dihydropyridine-3,5-dicarbonitrile) 6

Yield 79%, melting point 235.8°C. IR:υ_max_./cm^-1^ 3448, 3370 (2NH_2_),2939,2860 (CH aliph.), 2218 (2 C ≡ N), 1685 (2 C = O), 1390, 1149 (SO_2_). ^1^ H-NMR (DMSO-d_6,_ D_2_O):δ 6.6, 6.9 (2d, 4 H, 2 CH = CH, J = 7.4,7.3 Hz), 7.0-7.8 (m, 22 H, Ar-H + 2NH_2_, exchangable). ^13^ C-NMR(DMSO-d_6_): 62.9(2), 101.8(2), 113.0(4), 120.7(4), 125.7(2), 126.8(4), 128.1(2), 128.5(4), 129(4), 131.9(2), 133.7(2), 137.2(2), 138.8(2), 154.3(2), 155.9(2), 176.1(2). Anal. Calcd. for C_42_H_26_N_8_O_4_S(738.77): C, 68.28; H, 3.55; N, 15.17. Found: C, 68.53; H, 3.61; N, 14.92.

##### 1,1'-(4,4'-sulfonylbis(4,1-phenylene))bis(6-amino-4-(3-nitrophenyl)-2-oxo-1,2-dihydropyridine-3,5-dicarbonitrile) 7

Yield 81%, melting point 167.2°C. IR:υ_max_./cm^-1^ 3375, 3213 (2 NH_2_), 3093 (CH arom.), 2218 (2 C ≡ N), 1674 (2 C = O), 1350, 1149 (SO_2_), 1593, 1330 (NO_2_).^1^ H-NMR (DMSO-d_6,_ D_2_O):δ 6.6 (s, 4 H, 2NH_2_, exchangable), 7.5-8.4 (m, 16 H, Ar-H). ^13^ C-NMR(DMSO-d_6_): 75.6(2), 113.0(2), 116.7(4), 122.8(2), 124.8(4), 125.1(2), 128.8(4), 129.8(2), 134.5(2), 136.0(2), 137.2(2), 138.4(2), 147.7(2), 156.9(2), 159.1(2), 165.5(2). Anal. Calcd. for C_38_H_20_N_10_O_8_S(776.69): C, 58.76; H, 2.60; N, 18.03. Found: C, 58.90; H, 2.91; N, 17.89.

##### 1,1'-(4,4'-sulfonylbis(4,1-phenylene))bis(6-amino-4-(4-nitrophenyl)-2-oxo-1,2-dihydropyridine-3,5-dicarbonitrile) 8

Yield 72%, melting point 208.0°C. IR:υ_max_./cm^-1^ 3371, 3210 (2 NH_2_), 3100 (CH arom.), 2194 (2 C ≡ N), 1674 (2 C = O), 1346, 1149 (SO_2_), 1593, 1332 (NO_2_). ^1^ H-NMR (DMSO-d_6,_ D_2_O):δ 6.7 (s, 4 H, 2NH_2_, exchangable), 7.8-8.4 (m, 16 H, Ar-H). ^13^ C-NMR(DMSO-d_6_): 73.7(2), 114.4(2), 114.9(4), 124.5(4), 127.9(4), 129.1(4), 130.2(4), 137.6(2), 138.9(2), 141.1(2), 144.6(2), 156.8(2), 157.3(2), 166.9(2). Anal. Calcd. for C_38_H_20_N_10_O_8_S(776.69): C, 58.76; H, 2.60; N, 18.03. Found: C, 59.00; H, 2.78; N, 18.09.

##### 1,1'-(4,4'-sulfonylbis(4,1-phenylene))bis(6-amino-2-oxo-4-(2,3,4-trimethoxyphenyl)-1,2-dihydropyridine-3,5-dicarbonitrile) 9

Yield 70%, melting point 286.4°C. IR:υ_max_./cm^-1^ 3378, 3210 (2 NH_2_), 3097 (CH arom.), 2943, 2839(CH aliph.), 2218 (2 C ≡ N), 1674 (2 C = O), 1390, 1157 (SO_2_).^1^ H-NMR (DMSO-d_6,_ D_2_O):δ 3.7, 3.8 (2 s, 18 H, 6OCH_3_), 6.7 (s, 4 H, 2NH_2_, exchangable), 6.9-8.0 (m, 12 H, Ar-H). ^13^ C-NMR(DMSO-d_6_): 55.8(2), 55.9(2), 60.5(2), 76.7(2), 107.7(2), 113.3(2), 115.4(2), 116.1(4), 121.1(2), 124.9(4), 129.8(4), 130.6(2), 138.9(2), 141.8(2), 150.1 (2), 153.5(2), 156.4(2), 159.6(2), 165.9(2). Anal. Calcd. for C_44_H_34_N_8_O_10_S(866.85): C, 60.96; H, 3.95; N, 12.93. Found: C, 60.72; H, 4.03; N, 12.81.

##### 1,1'-(4,4'-sulfonylbis(4,1-phenylene))bis(6-amino-4-(benzo[d][1,3]dioxol-5-yl)-2-oxo-1,2-dihydropyridine-3,5-dicarbonitrile) 10

Yield 77%, melting point 291.6°C. IR:υ_max_./cm^-1^ 3313, 3197 (2 NH_2_), 3100 (CH arom.), 2912, 2836(CH aliph.), 2214 (2 C ≡ N), 1686 (2 C = O), 1390, 1195 (SO_2_).^1^ H-NMR (DMSO-d_6,_ D_2_O):δ 6.1 (s, 4 H, 2CH_2_), 6.3 (s, 4 H, 2NH_2_, exchangable), 7.0-8.0 (m, 14 H, Ar-H). ^13^ C-NMR(DMSO-d_6_): 79.1(2), 102.4(2), 109.1(2), 113.9(2), 115.7(2), 116.2(4), 120.5(2), 125.7(4), 128.3(2), 128.4(4), 136.0(4), 148.1(4), 151.2(2), 161.3(2), 164.9 (2). Anal. Calcd. for C_40_H_22_N_8_O_8_S(774.72): C, 62.01; H, 2.86; N, 14.46. Found: C, 61.88; H, 2.94; N, 14.30.

##### 1,1'-(4,4'-sulfonylbis(4,1-phenylene))bis(6-amino-4-(2-chlorophenyl)-2-oxo-1,2-dihydropyridine-3,5-dicarbonitrile)11

Yield 69%, melting point 251.4°C. IR:υ_max_./cm^-1^ 3380, 3213 (2 NH_2_), 3097 (CH arom.), 2222 (2 C ≡ N), 1678 (2 C = O), 1380, 1153 (SO_2_), 740 (C-Cl).^1^ H-NMR (DMSO-d_6,_ D_2_O):δ 6.6 (s, 4 H, 2NH_2_, exchangable), 7.4-7.9 (m, 16 H, Ar-H). ^13^ C-NMR(DMSO-d_6_): 76.2(2), 114.8(2), 115.4(4), 124.6(4), 128.7(2), 129.8(2), 130.1(4), 130.3(2), 130.6(2), 131.7(2), 133.8(2), 138.7(2), 141.9(2), 153.7(2), 156.7(2), 159.4(2). Anal. Calcd. for C_38_H_20_Cl_2_N_8_O_4_S(755.59): C, 60.40; H, 2.67; N, 14.83. Found: C, 60.32; H, 2.79; N, 14.61.

##### 1,1'-(4,4'-sulfonylbis(4,1-phenylene))bis(6-amino-4-(4-chlorophenyl)-2-oxo-1,2-dihydropyridine-3,5-dicarbonitrile)12

Yield 86%, melting point 313.1°C. IR:υ_max_./cm^-1^ 3387, 3329 (2 NH_2_), 3093 (CH arom.), 2187 (2 C ≡ N), 1660 (2 C = O), 1370, 1161 (SO_2_), 771 (C-Cl). ^1^ H-NMR (DMSO-d_6,_ D_2_O):δ 6.6 (s, 4 H, 2NH_2_, exchangable), 7.5-8.1 (m, 16 H, Ar-H). ^13^ C-NMR(DMSO-d_6_): 80.2(2), 114.2(2), 115.6(4), 123.6(4), 128.7(4), 128.9(4), 130.0(4), 130.1(4), 134.8(4), 158.8(2), 153.9(2), 163.4(2). Anal. Calcd. for C_38_H_20_Cl_2_N_8_O_4_S(755.59): C, 60.40; H, 2.67; N, 14.83. Found: C, 60.71; H, 2.38; N, 15.08.

##### 1,1'-(4,4'-sulfonylbis(4,1-phenylene))bis(6-amino-4-(4-(dimethylamino)phenyl)-2-oxo-1,2-dihydropyridine-3,5-dicarbonitrile)13

Yield 71%, melting point 277.4°C. IR:υ_max_./cm^-1^ 3464, 3367 (2 NH_2_), 3097 (CH arom.), 2908, 2870 (CH aliph.), 2210 (2 C ≡ N), 1678 (2 C = O), 1381, 1168 (SO_2_).^1^ H-NMR (DMSO-d_6,_ D_2_O):δ 3.0 (s, 12 H, 4 CH_3_), 6.8 (s, 4 H, 2NH_2_, exchangable), 7.5-8.0 (m, 16 H, Ar-H). ^13^ C-NMR(DMSO-d_6_): 40.1(4), 78.1(2), 112.9(4), 117.8(2), 118.4(4), 120.2(2), 125.8(4), 128.2(4), 129.2(4), 127.6(2), 143.2(2), 151.4(2), 153.4(2), 162.2(2), 162.3(2). Anal. Calcd. for C_42_H_32_N_10_O_4_S(772.83): C, 65.27; H, 4.17; N, 18.12. Found: C, 65.50; H, 4.00; N, 18.41.

##### 1,1'-(4,4'-sulfonylbis(4,1-phenylene))bis(6-amino-4-(2-methoxyphenyl)-2-oxo-1,2-dihydropyridine-3,5-dicarbonitrile)14

Yield 70%, melting point 269.6°C. IR:υ_max_./cm^-1^ 3448, 3367 (2 NH_2_), 3066 (CH arom.), 2935, 2870 (CH aliph.), 2183 (2 C ≡ N), 1678 (2 C = O), 1350, 1149 (SO_2_).^1^ H-NMR (DMSO-d_6,_ D_2_O):δ 3.9 (s, 6 H, 2 OCH_3_), 6.6 (s, 4 H, 2NH_2_, exchangable), 7.3-8.1 (m, 16 H, Ar-H). Anal. Calcd. for C_40_H_26_N_8_O_6_S(746.75): C, 64.34; H, 3.51; N, 15.01. Found: C, 64.77; H, 3.31; N, 15.36.

##### 1,1'-(4,4'-sulfonylbis(4,1-phenylene))bis(6-amino-4-(4-methoxyphenyl)-2-oxo-1,2-dihydropyridine-3,5-dicarbonitrile)15

Yield 76%, melting point 303.8°C. IR:υ_max_./cm^-1^ 3317, 3197 (2 NH_2_), 3070 (CH arom.), 2339, 2843 (CH aliph.), 2214 (2 C ≡ N), 1686 (2 C = O), 1370, 1149 (SO_2_).^1^ H-NMR (DMSO-d_6,_ D_2_O):δ 3.9 (s, 6 H, 2 OCH_3_), 6.3 (s, 4 H, 2NH_2_, exchangable), 7.0-8.3 (m, 16 H, Ar-H). ^13^ C-NMR(DMSO-d_6_): 56.0(2), 80.2(2), 105.4(2), 114.2(2), 115.6(4), 123.4(4), 123.8(4), 128.6(4), 131.3(4), 133.1(4), 133.6(2), 134.2(4), 134.8(2), 151.3(2), 152.8(2), 153.8(2), 163.4(2). Anal. Calcd. for C_40_H_26_N_8_O_6_S(746.75): C, 64.34; H, 3.51; N, 15.01. Found: C, 64.48; H, 3.70; N, 14.92.

##### 1,1'-(4,4'-sulfonylbis(4,1-phenylene))bis(6-amino-2-oxo-4-(thiophen-2-yl)-1,2-dihydropyridine-3,5-dicarbonitrile)16

Yield 81%, melting point 187.7°C. IR:υ_max_./cm^-1^ 3375, 3213 (2 NH_2_), 3100 (CH arom.), 2214 (2 C ≡ N), 1676 (2 C = O), 1390, 1149 (SO_2_).^1^ H-NMR (DMSO-d_6,_ D_2_O):δ 6.6 (s, 4 H, 2NH_2_, exchangable), 7.2-8.7 (m, 14 H, Ar-H). ^13^ C-NMR(DMSO-d_6_): 75.3(2), 116.2(4), 120.5(2), 127.2(2), 127.7(2), 128.7(2), 129.8(4), 130.4(2), 135.6(2), 138.1(2), 144.6(2), 153.5(2), 162.1(2), 171.9(2). Anal. Calcd. for C_34_H_18_N_8_O_4_S_3_(698.75): C, 58.44; H, 2.60; N, 16.04. Found: C, 58.19; H, 2.90; N, 16.32.

##### 1,1'-(4,4'-sulfonylbis(4,1-phenylene))bis(6-amino-2-oxo-1,2-dihydropyridine-3,5-dicarbonitrile)17

Yield 66%, melting point 308.9°C. IR:υ_max_./cm^-1^ 3371, 3206 (2 NH_2_), 2187 (2 C ≡ N), 1680 (2 C = O), 1377, 1145 (SO_2_).^1^ H-NMR (DMSO-d_6,_ D_2_O):δ6.8 (s, 4 H, 2NH_2_, exchangable), 7.6-7.8 (m, 10 H, Ar-H + 2CH pyridone). ^13^ C-NMR(DMSO-d_6_): 62.9(2), 100.2(2), 116.2(4), 122.9(4), 130.2(4), 136.9(2), 139.6(2), 151.7(2), 154.2(2), 158.7(2). Anal. Calcd. for C_26_H_14_N_8_O_4_S(534.51): C, 58.42; H, 2.64; N, 20.96. Found: C, 58.60; H, 2.88; N, 20.71.

##### 1,1'-(4,4'-sulfonylbis(4,1-phenylene))bis(6-amino-4-methyl-2-oxo-1,2-dihydropyridine-3,5-dicarbonitrile)18

Yield 68%, melting point 230.2°C. IR:υ_max_./cm^-1^ 3410, 3394 (2 NH_2_), 2935, 2860 (CH aliph.), 2198 (2 C ≡ N), 1686 (2 C = O), 1390, 1149 (SO_2_).^1^ H-NMR (DMSO-d_6,_ D_2_O):δ 1.6 (s,6 H, 2 CH_3_), 6.7 (s, 4 H, 2NH_2_, exchangable), 7.3-8.0 (m, 8 H, Ar-H). ^13^ C-NMR(DMSO-d_6_): 8.4(2), 56.2(2), 114.9(2), 116.2(4), 119.5(4), 128.1(4), 136.7(2), 136.9(2), 151.2(2), 152.8(2), 166.9(2). Anal. Calcd. for C_28_H_18_N_8_O_4_S(562.56): C, 59.78; H, 3.23; N, 19.92. Found: C, 59.54; H, 3.40; N, 19.69.

##### 1,1'-(4,4'-sulfonylbis(4,1-phenylene))bis(6-amino-4-butyl-2-oxo-1,2-dihydropyridine-3,5-dicarbonitrile)19

Yield 62%, melting point 236.3°C. IR:υ_max_./cm^-1^ 3380, 3367 (2 NH_2_), 2954, 2840 (CH aliph.), 2195 (2 C ≡ N), 1650 (2 C = O), 1399, 1149 (SO_2_).^1^ H-NMR (DMSO-d_6,_ D_2_O):δ 1.1 (t,6 H, 2 CH_3_), 1.3-2(m,12 H,6 CH_2_), 6.6 (s, 4 H, 2NH_2_, exchangable), 7.3-7.9 (m, 8 H, Ar-H). ^13^ C-NMR(DMSO-d_6_): 13.5(2), 21.7(2), 22.3(2), 29.1(2), 62.6(2), 113.5(2), 113.9(4), 120.8(4), 129.1(4), 133.9(2), 142.3(2), 153.6(2), 161.6(2), 175.8(2). Anal. Calcd. for C_34_H_30_N_8_O_4_S(646.72): C, 63.14; H, 4.68; N, 17.33. Found: C, 63.00; H, 4.90; N, 17.01.

##### (2E,2'E)-N,N'-(4,4'-sulfonylbis(4,1-phenylene))bis(2-cyano-3-(2,4-dichlorophenyl)acrylamide) 20

A mixture of 2 (3.82 g, 0.01 mol.) and 2,4-dichlorobenzaldehyde (3.50 g, 0.02 mol.) in acetic acid was refluxed for 8 h, the obtained solid was filtered and recrystallized from acetic acid to give 20. Yield 88%, melting point 253.4°C. IR:υ_max_./cm^-1^ 3372 (2 NH), 3986 (CH arom.), 2940, 2860 (CH aliph.), 2203 (2 C ≡ N), 1652 (2 C = O), 1390, 1145 (SO_2_), 829 (C-Cl).^1^ H-NMR (DMSO-d_6,_ D_2_O):δ6.9-7.8 (m, 14 H, Ar-H), 10.0 (s, 2 H, 2NH, exchangable). ^13^ C-NMR(DMSO-d_6_): 112.8(2), 119.0(2), 122.8(4), 127.9(2), 128.5(4), 129.0(2), 130.1(2), 130.4(2), 132.6(2), 134.6(2), 135.9(2), 142.8(2), 153.3(2), 163.3(2). Anal. Calcd. for C_32_H_18_C_l4_N_4_O_4_S(696.39): C, 55.19; H, 2.61; N, 8.05. Found: C, 55.36; H, 2.50; N, 7.99.

##### N,N'-(4,4'-sulfonylbis(4,1-phenylene))bis(2-oxo-2 H-chromene-3-carboxamide) 21

To a solution of 2 (3.82 g, 0.01 mol.) in acetic anhydride (30 mL), salicylaldehyde (2.44 g, 0.02 mol.) and fused Na acetate (1.6 g, 0.02 mol.) were added, the reaction mixture was refluxed for 3 h, cooled and the solid obtained was crystallized from dioxane to give 21. Yield 59%, melting point 195.5°C. IR:υ_max_./cm^-1^ 3433 (2 NH), 3097 (CH arom.), 1762, 1720 (4 C = O), 1396, 1157 (SO_2_).^1^ H-NMR (DMSO-d_6,_ D_2_O):δ 7.3-7.6 (m, 16 H, Ar-H), 8.2 (s, 2 H, 2CH), 10.4 (s, 2 H, 2NH, exchangable). ^13^ C-NMR(DMSO-d_6_): 118.9(2), 121.8(2), 123.3(2), 124.4(4), 126.4(2), 127.7(2), 128.8(2), 130.7(4), 137.6(2), 141.2(2), 144.3(2), 152.1(2), 169.1(2), 171.9(2). Anal. Calcd. for C_32_H_20_N_2_O_8_S(592.57):C, 64.86; H, 3.40; N, 4.73. Found: C, 64.76; H, 3.31; N, 5.00.

##### N,N'-(4,4'-sulfonylbis(4,1-phenylene))bis(2-imino-2 H-chromene-3-carboxamide) 22

A mixture of compound 2 (3.82 g, 0.01 mol.), salicylaldehyde (2.44 g, 0.02 mol.) and anhydrous ammounium acetate (2.30 g, 0.03 mol.) was refluxed in ethanol (50 mL) for 2 h. The solid obtained was crystallized from ethanol to give 22.Yield 64%, melting point 256.1°C. IR:υ_max_./cm^-1^ 3383, 3259 (4 NH), 1686 (2 C = O), 1377, 1149 (SO_2_).^1^ H-NMR (DMSO-d_6,_ D_2_O):δ 7.2-8.0 (m, 16 H, Ar-H), 8.6 (s, 2 H, 2CH), 10.4 (s, 2 H, 2NH, exchangable), 13.1 (s, 2 H, 2NH, exchangable),. ^13^ C-NMR(DMSO-d_6_): 114.9(2), 119.7(2), 122.2(2), 122.5(4), 125.7(2), 127.9(2), 128.8(2), 129.4(4), 134.1(2), 141.6(2), 142.1(2), 153.7(2), 160.2(2), 165.5(2). Anal. Calcd. for C_32_H_22_N_4_O_6_S(590.61): C, 65.08; H, 3.75; N, 9.49. Found: C, 65.23; H, 3.60; N, 9.29.

#### General procedure for synthesis of compound 23 and 24

Equimolar amount of compound 21or 22 (0.01 mol.) and malononitrile (1.32 g, 0.02 mol.) and anhydrous ammonium acetate (2.30 g, 0.03 mol.) were refluxed in ethanol (50 mL) for 5 h. The obtained solid was crystallized from dioxane to give *23 and 24*, respectively.

##### 3,3'-(4,4'-sulfonylbis(4,1-phenylene))bis(2-amino-4,5-dioxo-4,5-dihydro-3 H-chromeno[3,4-c]pyridine-1-carbonitrile) 23

Yield 75%, melting point 267.8°C. IR:υ_max_./cm^-1^ 3444, 3344 (2 NH_2_), 3100 (CH arom.), 2199 (2 C ≡ N), 1690, 1660 (4 C = O), 1373, 1153 (SO_2_). ^1^ H-NMR (DMSO-d_6,_ D_2_O):δ 6.7 (s, 4 H, 2NH_2_, exchangable), 7.1-8.0 (m, 16 H, Ar-H). ^13^ C-NMR(DMSO-d_6_): 76.7(2), 115.8(2), 119.0(2), 121.8(2), 122.6(4), 123.9(2), 124.8(2), 128.3(4), 129.0(2), 135.0(4), 141.1(2), 143.5(2), 158.0(2), 158.4(2), 164.8(2), 169.1(2), 170.3(2). Anal. Calcd. for C_38_H_20_N_6_O_8_S(720.67): C, 63.33; H, 2.80; N, 11.66. Found: C, 63.11; H, 2.96; N, 11.49.

##### 3,3'-(4,4'-sulfonylbis(4,1-phenylene))bis(2-amino-5-imino-4-oxo-4,5-dihydro-3 H-chromeno[3,4-c]pyridine-1-carbonitrile) 24

Yield 77%, melting point >360°C. IR:υ_max_./cm^-1^ 3441, 3348, 3236, 3186 (2NH, 2NH_2_), 2203 (2 C ≡ N), 1680 (2 C = O), 1381, 1153 (SO_2_).^1^ H-NMR (DMSO-d_6,_ D_2_O):δ 6.6 (s, 4 H, 2NH_2_, exchangable), 7.1-7.9 (m, 16 H, Ar-H), 8.9(s, 2 H, 2NH, exchangable). ^13^ C-NMR(DMSO-d_6_): 78.1(2), 112.6(2), 116.7(4), 122.4(2), 122.8(2), 124.6(4), 128.1(2), 128.9(4), 129.1(2), 137.4(2), 155.6(2), 156.1(2), 158.7(2), 159.6(2), 163.5(2). Anal. Calcd. for C_38_H_22_N_8_O_6_S(718.70):C, 63.50; H, 3.09; N, 15.59. Found: C, 63.44; H, 3.18; N, 15.75.

#### Molecular docking

All the molecular modeling studies were carried out on an Intel Pentium 1.6 GHz processor, 512 MB memory with Windows XP operating system using Molecular Operating Environment (MOE, 10.2008) software. All the minimizations were performed with MOE until a RMSD gradient of 0.05 kcal mol^-1^A^o-1 ^with MMFF94X force field and the partial charges were automatically calculated. The X-ray crystallographic structure of franesyltransferase and arginine methyltransferase (PRMT1) complexes with their ligands (PDB ID: 3E30, 3Q7E) were obtained from the protein data bank. The enzymes were prepared for docking studies where: (i) Ligand molecule was removed from the enzyme active site. (ii) Hydrogen atoms were added to the structure with their standard geometry. (iii) MOE Alpha Site Finder was used for the active sites search in the enzyme structure and dummy atoms were created from the obtained alpha spheres. (iv) The obtained model was then used in predicting the ligand enzymes interactions at the active site.

#### *In vitro* antitumor activity

Human tumor breast cell line (MCF7) was used in this study. The cytotoxic activity was measured *in vitro* for the newly synthesized compounds using the Sulfo-Rhodamine-B stain (SRB) assay using the method of Skehan et al. [52]. The *in vitro* anticancer screening was done by the pharmacology unit at the National Cancer Institute, Cairo University.

Cells were plated in 96-multiwell plate (104 cells/well) for 24 h before treatment with the compound(s) to allow attachment of cell to the wall of the plate. Test compounds were dissolved in dimethyl sulfoxide. Different concentrations of the compound under test (10, 25, 50, and 100 μM) were added to the cell monolayer. Triplicate wells were prepared for each individual concentration. Monolayer cells were incubated with the compound(s) for 48 h at 37°C and in atmosphere of 5% CO_2_. After 48 h, cells were fixed, washed and stained for 30 min with 0.4% (wt/vol) SRB dissolved in 1% acetic acid. Excess unbound dye was removed by four washes with 1% acetic acid and attached stain was recovered with Trise-EDTA buffer. Color intensity was measured in an ELISA reader. The relation between surviving fraction and drug concentration is plotted to get the survival curve for breast tumor cell line after the specified time. The molar concentration required for 50% inhibition of cell viability (IC_50_) was calculated and compared to the reference drug Doxorubicin (CAS, 25316-40-9). The surviving fractions were expressed as means ± standard error and the results are given in Table 3.

## Conclusions

Diarylsulfone derivatives may serve as good candidates in the search for novel anticancer agents as illustrated by the IC_50_ values of the investigated compounds. These values were better than that of Doxorubicin. The mechanism of action as anticancer of the synthesized compounds was investigated through molecular docking on the active site of farnesyl transferase and arginine methyltransferase. Both enzymes could be the target of action of these compounds based on the good energy scores and amino acid interactions in the active sites of enzymes however, the exact mechanism of action still needs more investigation to be clarified.

## **Competing interests**

The authors declare that they have no competing interests.

## **Authors' contributions**

M.Al-Said, M.Ghorab designed the synthetic schemes for all synthesized compounds. All authors contributed in the chemical synthesis. Y.Nissan carried out molecular docking and interpretation of its results as well as interpretation of the biological results. All authors read and approved the final manuscript. All authors read and approved the final manuscript.
